# Anti-Inflammatory and Analgesic Activities of the Saponin, Daucosterol, and the Triterpenoid Ester, *β*-Sitosterol 3-Myristate, From *Capparis erythrocarpos* (Isert) Capparaceae, and Their Interaction With the TRPV1 Ion Channel Transporter

**DOI:** 10.1155/bri/2621242

**Published:** 2025-09-04

**Authors:** Emmanuel Kofi Kumatia, Nguyen Huu Tung, Alex Asase

**Affiliations:** ^1^Department of Phytochemistry, Centre for Plant Medicine Research, Mampong, Akuapem, Ghana; ^2^Department of Medicinal Chemistry and Drug Quality Control, Faculty of Pharmacy, Phenikaa University, Hanoi, Vietnam; ^3^Plant Development Department, Centre for Plant Medicine Research, Mampong, Akuapem, Ghana

**Keywords:** carrageenan, daucosterol, saponin, transient receptor potential vanilloid 1 (TRPV1), triterpenoid

## Abstract

*Capparis erythrocarpos* is a medicinal plant traditionally used as an antiarthritic, anti-inflammatory, and analgesic agent. However, no previous reports exist on the analgesic and acute anti-inflammatory activities of the individual chemical constituents from this plant. This study reports the isolation, characterization, and evaluation of analgesic and acute anti-inflammatory activities of chemical constituents from the root bark of *C. erythrocarpos*, including their modulatory effects on TRPV1 ion channel activity. The isolated compounds were characterized as daucosterol (DC) and *β*-sitosterol 3-myristate (SM) using NMR and LC/GC-MS. DC significantly (*p* < 0.05) inhibited both phases of carrageenan-induced edema in a reverse dose-dependent manner, demonstrating 58.38% anti-inflammatory activity at 2 mg/kg p.o., comparable to diclofenac sodium (DS) at 8 mg/kg p.o. (53.07%). SM inhibited only phase 2 of carrageenan-induced edema. Both compounds significantly inhibited cold-induced pain with superior analgesic activity compared to DS. Against inflammation-induced pain, DC showed the highest analgesic activity (47.37% at 2 mg/kg p.o.). Molecular docking studies revealed that DC and SM produced ΔG values of −10.60 and −9.80 kcal/mol, respectively, which are more negative than those of DS (−8.6 kcal/mol), suggesting that they might be superior TRPV1 ion channel inhibitors and that DS likely has additional mechanisms of action. These results demonstrate that DC and SM possess remarkable therapeutic properties, warranting further exploration for novel drug development.

## 1. Introduction

Pain is a vital sensation signaling tissue damage triggered by internal or external harmful occurrences [1]. Inflammation of a wounded tissue or injury to the nerve in a damaged tissue generates enhanced input from nociceptive fibers to create hyperexcitable conditions among nociceptive pathways in the CNS. This then leads to hyperalgesia, allodynia, and spontaneous pain [2].

Cold temperatures are one of the harmful stimuli that cause significant, irreparable tissue injury, and painful sensation [3]. Detection of cold or low temperature is, therefore, crucial for survival in the animal kingdom. Hence, various animals have developed numerous approaches to mitigate, circumvent, and/or escape cold conditions [4].

Various classes of drugs currently available for treating pain and inflammatory diseases—including opioids, nonsteroidal anti-inflammatory drugs (NSAIDs), anticonvulsants, antidepressants, and cannabinoids—produce severe adverse effects [5, 6]. In most cases, these adverse effects outweigh the therapeutic benefits. Therefore, there is a critical need to develop novel, potent analgesics that have fewer adverse effects.

Ion channels are widely distributed throughout vertebrate tissues and are involved in numerous biological processes in both excitable and nonexcitable cells. Transient receptor potential (TRP) ion channels constitute a family of nonselective cation channels consisting of 28 different members divided into six subfamilies, including TRP vanilloids (TRPV) [7]. The TRPV subfamily comprises six members (TRPV1–6). Among these, TRPV1 channels are predominantly located in neurons associated with pain perception and exhibit a remarkable range of activation mechanisms, voltage dependence, selectivity, and pharmacological properties that distinguish them from other ion channel families [8]. These distinctive characteristics of TRPV1 ion channels make them attractive targets for developing novel therapeutic agents for treating pain, inflammatory diseases, and cancer [9, 10].

*Capparis erythrocarpos*, a medicinal plant in the Capparaceae family, is a shrub with obovate to elliptic, ovate, and lanceolate leaves that have obtuse tips and are curved at the base. The leaves, which are 3–14 cm long and 1.5–5.0 cm wide, have surfaces covered with clusters of small thorns [11, 12]. In African traditional medicine, the whole root or root bark is the primary part used for treatment and is the most extensively studied morphological component of the plant. The root of *C. erythrocarpos* is prepared in various dosage forms to treat conditions such as pain, inflammation, arthritis, blurred vision, and abdominal gas in traditional medicine [12, 13]. The plant has been reported to demonstrate pharmacological activities including analgesic, anti-inflammatory, antiarthritic, and antimalarial effects [11, 14, 15].

So far, *β*-sitosterol, *β*-sitosterol 3-myristate (SM), and *β*-sitosterol 3-palmitate were isolated from the root bark of *C. erythrocarpos* and were shown to inhibit CFA-induced arthritis in rats [15]. Betulinic acid was also isolated from the leaf and its antimalarial activity elucidated [14]. However, the anti-inflammatory activity of the chemical constituents of *C. erythrocarpos*' root against acute inflammation and pain is not known. The aim of this study is, therefore, to isolate and characterize the chemical constituents of the root bark of *C. erythrocarpos* and to evaluate their anti-inflammatory and analgesic activities in laboratory rats.

## 2. Materials and Methods

### 2.1. Chemicals

Analytical-grade solvents such as petroleum ether (40°C–60°C), chloroform (CF), and ethyl acetate were procured from Fisher Scientific (Loughborough, U.K.) and Park Scientific Limited (Northampton, U.K.), respectively. Food-grade ethanol (99%) was supplied by Midland Ghana Limited (Tema). Silica gel (particle size 40–60 μm and 230 × 400 mesh size) and TLC plate (20 × 20 cm aluminum sheet framework coated with TLC silica gel 60 F_254_) were acquired from Sorbent Technologies (Atlanta, GA, USA) and Merck Chemicals (Darmstadt, Germany), respectively. Diclofenac sodium (DS) (Research grade) was obtained from Sigma Chemical Co. (St. Louis, USA).

### 2.2. Collection, Extraction, and Partitioning of *C. erythrocarpos* Root Bark

Collection, extraction, and isolation of compounds from the root bark of *C. erythrocarpos* were carried out using our previous methods [15]. *C. erythrocarpos* root was harvested in January 2017 from Dodowa in the Shai Osudoku District of the Greater Accra Region of Ghana. The plant sample was assigned voucher number CPMR 4876 and deposited in the herbarium of CPMR [11]. The root was cleaned with water, and the bark was removed, cut into fragments, sun-dried for 10 days, and milled. The coarse root bark powder (264.2 g) was extracted with 70% ethanol (2 × 625 mL) for 6 h using a Soxhlet apparatus. The extraction process was repeated 12 times with 264.2 g of material each time, yielding a total extracted material of 3.17 kg. Ethanol was removed from the extract at 45°C using a rotary evaporator. The resulting aqueous mixture was divided into four equal portions and successively partitioned with equal volumes of petroleum ether (0.663 L × 3) and ethyl acetate (0.663 L × 3). The petroleum ether and ethyl acetate fractions were separately combined and concentrated using a rotary evaporator to obtain 10.28 g of Capparis root petroleum ether fraction (CRPF) and 6.78 g of Capparis root ethyl acetate fraction (CREF), respectively.

### 2.3. Purification of Petroleum Ether Fraction (CRPF)

Approximately 10.28 g of CRPF was mixed with silica gel and dried overnight. The mixture was transferred onto 225.00 g of normal-phase silica gel packed in a glass column and successively eluted with PE, and then, PE–CF mixture with CF being added incrementally at 5% until 100% CF was attained. CF-EtOH mixtures were then applied with EtOH being added incrementally at 5% until 100% EtOH was attained. The individual fractions collected were combined separately into 6 groups (CRPF1–CRPF6) using the similarities in their TLC profiles [15].

CRPF3 (536.6 mg), an orange semisolid, was further purified over 26.21 g wet silica gel in a small glass column. The column was eluted with 100 mL each of the following solvent systems: PE-CF 4:1; 7:3; 3:2; followed by CF-PE 1:1 (350 mL); CF 100% (200 mL); CF-EtOH 9:1 (200 mL). The fractions collected (68) were grouped into 3 (CRPF3 S1-S3) using their TLC profiles. CRPF3 S2, eluted with 100% CF, gave compound [**1**] [15].

### 2.4. Purification of the Ethyl Acetate Fraction (CREF)

CREF (6.78 g) was mixed with 23 g of silica gel and dried. A similar method was applied to purify the dried sample as described above for the petroleum ether fraction CRPF to obtain CRPF1-CRPF6. The 150 fractions collected were grouped into 5 major fractions CREF1-CREF5 [15]. Compound [**2**] was obtained from CREF4.

### 2.5. Development of TLC Chromatograms of the Compounds

The compounds were dissolved in CF, and the solutions were spotted on a normal-phase TLC plate on which the baseline and the solvent front were marked with parallel pencil lines. The plates were developed in the appropriate solvent system, stained with 10% H_2_SO_4,_ and heated at 80°C to develop the chromatograms.

### 2.6. NMR Analysis of the Compounds

A Bruker FT-NMR spectrometer (Avance 500 MHz, Germany) coupled with Bruker Topspin 3.6.3 NMR data analysis software was employed to acquire the NMR data (^1^H, ^13^C, ^1^H-^1^H COSY, DEPT-135, and HMBC) of the compounds at room temperature. Tetramethylsilane was used as the internal standard, and chemical shift values are given in *δ* (ppm). The compounds were dissolved in CDCl_3_, and the resulting solutions were used for the analysis [17].

### 2.7. Identification of Carboxylic Acid Moiety

The carboxylic acid moiety of [**1**] was identified using the process of saponification and derivatization of carboxylic acids from the esters as described by Kumatia et al. [15]. Briefly, around 2 mg of [**1**] was solubilized in methanol-sodium hydroxide solution (2 M) and heated for 1 h at 60°C over a water bath. The mixture was then removed from the water bath, 20 mL of distilled water was added, and it was allowed to cool to room temperature. It was thereafter extracted with 30 mL of n-hexane. The aqueous fraction was then acidified with 5 mL of 1 M HCl before extraction with 20 mL of ethyl acetate. The resulting ethyl acetate fraction was concentrated to 3 mL, and approximately 2 mL was transferred into a GC vial and analyzed using GC-MS (Agilent 7890B) equipped with an Agilent GC Sampler 80 and an Agilent 7000C Triple Quadrupole GC/MS, under the same conditions described in our previous study. The carboxylic acid residue of [1] was identified based on its mass fragmentation pattern and comparison with the NIST mass spectra database [15].

### 2.8. Pharmacological Studies

#### 2.8.1. Experimental Animals and Administration of Test Substances

Thirty female Wistar rats (88–129 g) in 6 groups (*N* = 5) were used for the study. The rats were housed in aluminum cages under controlled temperature and pressure and given distilled water and commercial pellet chow *ad libitum*. The experiments were conducted according to institutional, national, and international standards. Ethical clearance for the work was obtained from the Centre for Plant Medicine Institutional Review Committee with the permit number CPMR/M5-PT2/2022. Administration of the compounds was conducted as follows: Groups 1–2 received daucosterol (DC) 2 and 8 mg/kg p.o., respectively. Groups 3–4 received 2 and 8 mg/kg p.o. of *β*-sitosterol myristate. Group 5 received DS powder (8 mg/kg p.o.) as the standard control, while Group 6 received 1 mL of distilled water as the sham control.

#### 2.8.2. Evaluation of Anti-Inflammatory Activity

The baseline paw widths (Lo) of the rats were measured using a digital caliper (Fisherbrand Traceable, Cat. No. 14–648-17, Fisher Scientific, China) before any treatment. Approximately 10 min after the measurements, the various treatment agents were administered to the rats by oral gavage in 2% Tween 80 solution as a vehicle. Thereafter, the inflammation was induced according to Winter et al. [18] via subplantar injection of 0.2 mL of 1% carrageenan in normal saline into the right hind paw of each rat. The paw width (Lt) of each rat was subsequently measured at 1, 2, 3, and 4 h after the carrageenan injection. The anti-inflammatory activity of the compounds was calculated as follows:

(1) $$\begin{array}{c} \text{Ai}=\frac{\text{Lt}-\text{Lo}}{\text{Lo}}\;x\;100\%. \end{array}$$

#### 2.8.3. Evaluation of Inflammatory Pain Using the Mechanical Hyperalgesia Test

At the 4th hour after initiation of carrageenan-induced edema in the anti-inflammatory test above, the rats were restrained with the left hand, and the inflamed paws were gently pressed five consecutive times using the fingers of the right hand as described in the preprint of this paper (https://www.researchsquare.com/article/rs-6232211/v1). The number of vocalizations and/or withdrawal responses of the inflamed paw was recorded as an indicator of pain. The analgesic activity (Aa) of each group was calculated relative to the negative control using the following formula:

(2) $$\begin{array}{c} \text{Aa}=\frac{\text{Mean number of pain expressed by }\left(\text{treated group}-\text{control group}\right)}{\text{Mean number of pain expressed by control group}}\;x\;100\%. \end{array}$$

### 2.9. Cold Pain Test

Thirty female Wistar rats (*N* = 5), grouped similarly to the anti-inflammatory assay in 2.8.1, were used for this study. The study was conducted by adopting the method of Zhao et al. [19] with some modifications. Instead of a cold/plate, the rats were placed on ice blocks maintained at 1.50 ± 0.5°C. The reaction time (Tt) in seconds for paw licking, lifting, shaking, or jumping was recorded. The baseline reaction time (To) was determined 1 h before treatment with the drugs. The postdrug administration reaction time was determined at 1, 2, 3, and 4 h for each group. The analgesic activity (Aa) was calculated as

(3) $$\begin{array}{c} \text{Aa}=\frac{\text{Tt}-\text{To}}{\text{To}}\;x\;100\%. \end{array}$$

### 2.10. Docking Studies

Molecular docking studies of *β*-sitosterol myristate [**1**], DC [**2**], and DS were evaluated by employing a cavity-detection guided blind docking approach with CB-Dock2 [14, 20]. The PDB structure of the TRPV1 ion channel transporter protein 3j5p target protein was obtained from https://www.rcsb.org (RCSB PDB, 1971). Biova Discovery Studio 2023 was employed for visualization. Docking was initiated after water molecules, and het atoms were removed from the protein.

### 2.11. Statistical Analysis

GraphPad Prism statistical software Version 6 was used to analyze the data generated. Two-way ANOVA followed by Tukey's Post hoc test was used to analyze time-dependent effects, whereas one-way ANOVA followed by the Dunnett multiple comparison test was employed to analyze overall effects that were not time-dependent. Statistical significance was defined as *P* < 0.05.

## 3. Results

### 3.1. Characterization of the Compounds

#### 3.1.1. Structure Identification of Compound [**1**]

Compound **1** was obtained as an orange solid (93.4 mg), which was soluble in CF. R_f_ = 0.6944 (petroleum ether–ethyl acetate, 10:1). ^1^H NMR (CDCl_3_, 500 MHz), *δ*1.40 (2H, m, H-1), 1.5 (2H, m, H-2), 3.60 (1H, m, H-3), 5.48 (2H, m, H-4), 5.48 (1H, t, H-6), 2.05 (2H, m, H-7), 1.65 (1H, s, H-8), 1.6 (1H, s, H-9), 1.50 (2H, H-11), 1.5 (2H, m, H-12), 1.5 (1H, m, H-14), 1.60 (2H, m, H-15), 1.85 (2H, m, H-16), 1.50 (1H, m, H-17), 0.7 (2H, s, H-18), 1.00 (3H, s, H-19), 1.6 (1H, m, H-20), 0.90 (3H, d, H-21), 0.9 0.90 (2H, m, H-22), 0.90 (2H, m, H-23), 1.3 (1H, m, H-24), 1.6 (1H, m, H-25), 0.8 (3H, d, H-26), 0.90 (3H, d, H-27), 1.00 (2H, m, H-28), 0.80 (3H, t, H-29). 7.30 (1H, s, H-2′), 1.6 (1H, s, H-3′), 1.35 (1H, s, H-4′), 1.35 (1H, s, H-5′), 1.35 (1H, s, H-6′), 1.35 (1H, s, H-8′), 1.35 (1H, s, H-9′), 1.35 (1H, s, H-10′), 1.35 (1H, s, H-11′), 1.3 (1H, s, H-12′), 1.20 (1H, s, H-13′), and 0.90 (1H, s, H-14′). ^13^C NMR (CDCl_3_, 500 MHz), *δ* 37.25 (C-1), 31.93 (C-2), 71.86 (C-3), 42.21 (C-4), 140.72 (C-5), 121.74 (C-6), 31.93(C-7), 31.93 (C-8), 50.15 (C-9), 36.51 (C-10),19.81 (C-11), 39.79 (C-12), 42.23 (C-13), 56.78 (C-14), 24.71 (C-15), 28.25 (C-16), 56.07 (C-17), 14.11 (C-18), 19.39(C-19), 36.15 (C-19), 36.15 (C-20), 18.78(C-21), 33.97 (C-22), 26.11 (C-23), 45.85(C-24), 29.70 (C-25), 19.81 (C-26), 19.04 (C-27), 23.08 (C-28), 14.11 (C-29), 179.0 (C-1′), 31.93 (C-2′), 29.70 (C-3′), 29.70 (C-4′), 29.70 (C-5′), 29.70 (C-6′), 29.70 (C-7′), 29.70 (C-8′), 29.70 (C-9′), 29.70 (C-10′), (C-11′), 24.71 C-12′), 22.69 (C-13′), and 14.11 (C-14′) [15].

#### 3.1.2. GC-MS Analysis of Compounds [**1**]

**Figure 1:**
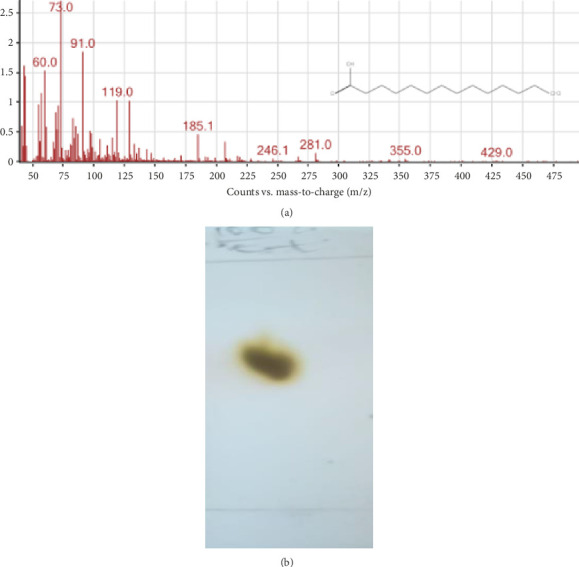
Structure of the carboxylic acid derivative of [**1**] and its GC-MS spectrum retrieved from the NIST database (a) and TLC chromatogram of [**2**], run in ethanol on a normal-phase TLC plate and heated at 80°C (b).

The GC-MS spectrum of the carboxylic acid moiety of [**1**] is shown in Figure 1. The RT of the carboxylic acid derivative of [**1**] was 12.91 min, and it was recognized in the NIST database to be tetradecanoic acid, also called myristic acid. The structure of [**1**] was confirmed as *β*-sitosterol 3-tetradecanoate (SM) [15].

#### 3.1.3. Structure Identification of Compound [**2**]

**Figure 2:**
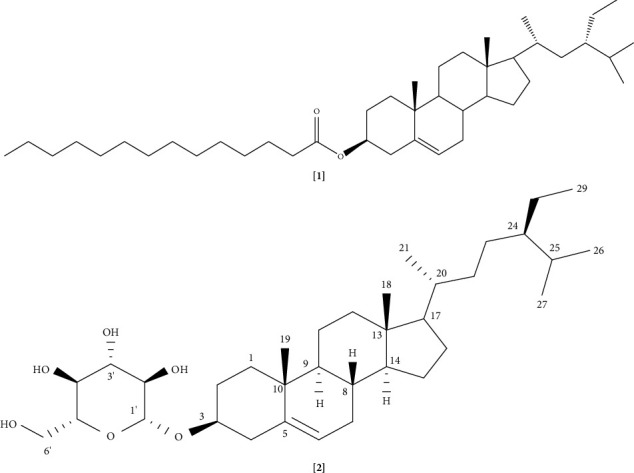
Chemical structures of *β*-sitosterol 3-myristate [**1**] and daucosterol [**2**] isolated from *C. erythrocarpos* root bark. These structures were deduced from the NMR and ESI-MS spectra of the compounds.

Compound **2** was obtained as an oyster white powder and gave a positive Liebermann–Burchard reaction; UV absorption was observed at 254 and 366 nm. The ^1^H-NMR spectrum of [**2**] revealed the presence of an olefinic proton at *δ*_*H*_ 5.37 (1H, m, H-6), a hydroxymethylene group at *δ*_*H*_ 3.60 (1H, m, H-3), 6 methyl groups, including 2 singlet signals at *δ*_*H*_ 0.71 (3H, s, H-18) and 1.03 (3H, s, H-19), 3 doublet signals at *δ*_*H*_ 0.94 (3H, d, *J* = 6.6 Hz, H-21), 0.83 (3H, d, *J* = 6.6 Hz, H-26), and 0.85 (3H, d, *J* = 6.6 Hz, H-27), and a triplet signal at *δ*_*H*_ 0.86 (3H, t, *J* = 7.8 Hz, H-29), indicating that it had a sterol structure. Furthermore, the compound exhibited signals characteristic of a *β*-D-glucopyranose moiety, including the anomeric proton [*δ*_*H*_ 4.40 (1H, d, *J* = 7.8 Hz, H-1′)], a hydroxymethylene group [*δ*_*H*_ 3.76 (1H, dd, *J* = 5.4, 12.0 Hz, H-6′) and 3.86 (1H, dd, *J* = 3.0, 12.0 Hz, H-6′)], and four hydroxymethylene groups [*δ*_*H*_ 3.20 (1H, m, H-2′), 3.38 (2H, m, H-3′, H-4′), and 3.27 (1H, m, H-5′)], suggesting a sterol glycoside. In addition, the ^13^C NMR spectrum of [**2**] revealed 35 carbon signals including two olefinic carbons at *δ*_C_ 140.4 (C-5) and 122.3 (C-6), one oxymethine at *δ*_C_ 79.3 (C-3), and an array of six carbons of a glucose unit [*δ*_C_ 101.2 (C-1′, Glc-1), 75.9 (C-2′, Glc-2), 77.3 (C-3′, Glc-3), 70.2 (C-4′, Glc-4), 77.7 (C-5′, Glc-5), and 61.9 (C-6′, Glc-6)], which were characteristic for stigmasterol glucoside. Additionally, the ESI-MS showed a quasimolecular ion peak at m/z 577.1 [M + H]^+^ consistent with the molecular formula of C_35_H_60_O_6_ (MW = 576). Based on the above evidence, the structure of [**2**] was identified as DC (Figure 2).

Compound 2: R_f_ = 0.5952 (100% absolute ethanol); mp = 285–287°C; [*α*]_*D*_^25^ = −40 (c 0,5, CDCl3) ESI-MS: *m/z* 577.1 [M + H]+; ^1^H-NMR (500 MHz, CDCl_3_): *δ*_*H*_ 3.60 (1H, m, H-3), 5.37 (1H, m, H-6), 0.71 (3H, s, H-18), 1.03 (3H, s, H-19), 0.94 (3H, d, *J* = 6.6 Hz, H-21), 0.83 (3H, d, *J* = 6.6 Hz, H-26), 0.85 (3H, d, *J* = 6.6 Hz, H-27), 0.86 (3H, t, *J* = 7.8 Hz, H-29), Glc: 4.40 (1H, d, *J* = 7.8 Hz, H-1′), 3.38 (2H, m, H-3′, H-4′), 3.27 (1H, m, H-5′), 3.20 (1H, m, H-2′), 3.76 (1H, dd, *J* = 5.4, 12.0 Hz, H-6′), 3.86 (1H, dd, *J* = 3.0, 12.0 Hz, H-6′); ^13^C NMR (125 MHz, CDCl_3_): δ_C_ 37.4 (C-1), 28.4 (C-2), 79.3 (C-3), 42.5 (C-4), 140.4 (C-5), 122.3 (C-6), 32.0 (C-7), 31.9 (C-8), 50.3 (C-9), 36.8 (C-10), 21.1 (C-11), 38.8 (C-12), 42.5 (C-13), 56.9 (C-14), 24.4 (C-15), 28.4 (C-16), 56.2 (C-17), 11.9 (C-18), 19.4 (C-19), 36.3 (C-20), 18.9 (C-21), 34.1 (C-22), 26.2 (C-23), 46.0 (C-24), 29.3 (C-25), 19.9 (C-26), 19.1 (C-27), 23.2 (C-28), 12.0 (C-29), 101.2 (C-1′, Glc-1), 75.9 (C-2′, Glc-2), 77.3 (C-3′, Glc-3), 70.2 (C-4′, Glc-4), 77.7 (C-5′, Glc-5), 61.9 (C-6′, Glc-6). The NMR data compare with those obtained for DC [21].

### 3.2. Anti-Inflammatory Activity

**Figure 3:**
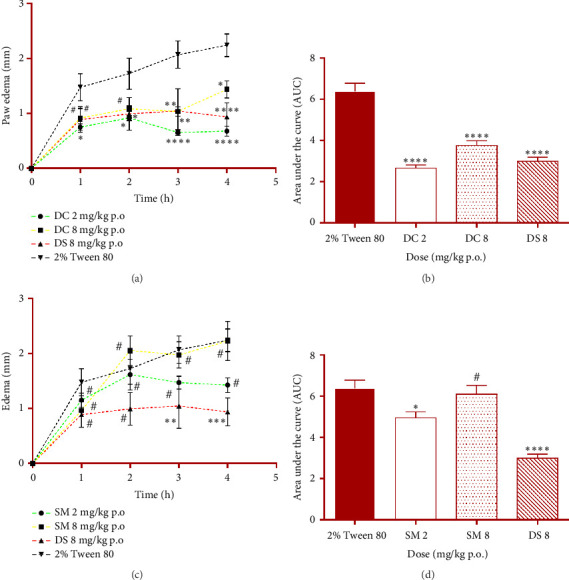
Effect of daucosterol (DC), *β*-sitosterol 3-myristate (SM), and diclofenac sodium (DS) on carrageenan-induced edema in rats' paw on time-course curves (a and c) and overall edema response calculated as net area under the curve (b and d). ^∗^*p* < 0.05; ^∗∗^*p* < 0.01; ^∗∗∗^*p* < 0.001; ^∗∗∗∗^*p* < 0.0001; or ^#^*p* > 0.05, (*N* = 5), compared to 2% Tween 80. Two-way ANOVA followed by Tukey's Post hoc test for the time-course curve or one-way ANOVA followed by the Dunnett multiple comparison test for the overall edema response.

The effects of administration of DC and SM on carrageenan-induced paw edema in rats are shown in Figure 3.

The inhibitory effect of DC 2 mg/kg p.o. on the carrageenan-induced edema commenced from the first hour after its administration (*p* < 0.05) and got more pronounced through the second to the fourth hour (*p* < 0.05 to 0.0001) on the time-course curve (Figure 3(a)). DC produced inverse dose-dependent inhibition of carrageenan-induced edema in rats' paw. SM also elicited an inverse dose-dependent inhibition of the carrageenan-induced edema in rats' paw. However, the effect was statistically insignificant (*p* > 0.05) from the first to the fourth hour at both doses (Figures 3(c) and 3(d)). On the other hand, the overall anti-inflammatory response shows that SM at 2 mg/kg p.o. produced significant (*p* < 0.05) anti-inflammatory activity. The anti-inflammatory effect of SM was less pronounced than that of DC. The anti-inflammatory activity produced by DC at 2 and 8 mg/kg p.o. was 58.38% and 41.28%, respectively. SM at 2 and 8 mg/kg p.o. also produced anti-inflammatory activity of 22.57% and 4.49%, respectively. The anti-inflammatory activity of the standard drug, DS, was 53.07%.

### 3.3. Analgesic Activity of DC and SM

#### 3.3.1. Effect of DC and SM on Inflammatory Pain

**Figure 4:**
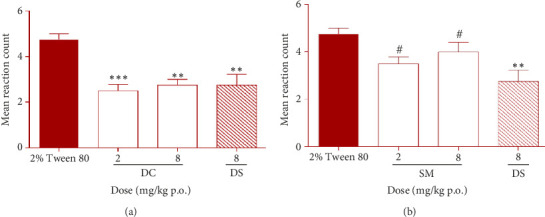
Effect of daucosterol (DC) (a), *β*-sitosterol 3-myristate (SM) (b), and diclofenac sodium (DS) on mechanical hyperalgesia in rats' paw during carrageenan-induced inflammation on the fourth hour. ^∗∗^*p* < 0.01; ^∗∗∗^*p* < 0.001, or ^#^*p* > 0.05, (*N* = 5), compared to 2% Tween 80 (one-way ANOVA followed by the Dunnett multiple comparison test).

The effects of DC and SM on the inhibition of hyperalgesia instigated by carrageenan-induced edema in rats' paws on the fourth hour are shown in Figures 4(a) and 4(b), respectively.

Both DC and SM inhibited the mean reaction count in rats in a dose-dependent manner. The effect was significant (*p* < 0.05) for DC at 2 and 8 mg/kg p.o. but statistically insignificant for SM at the same doses. The standard drug DS also produced significant (*p* < 0.05) inhibition of carrageenan-induced hyperalgesia with 42.11% analgesic activity (Figure 4). DC at 2 and 8 mg/kg p.o. inhibited carrageenan-induced hyperalgesia with analgesic activities of 47.37% and 42.11%, respectively. SM at 2 and 8 mg/kg p.o. produced analgesic activities of 26.32% and 15.79%, respectively.

#### 3.3.2. Effect of DC and SM on Ice-Induced Cold Pain

**Figure 5:**
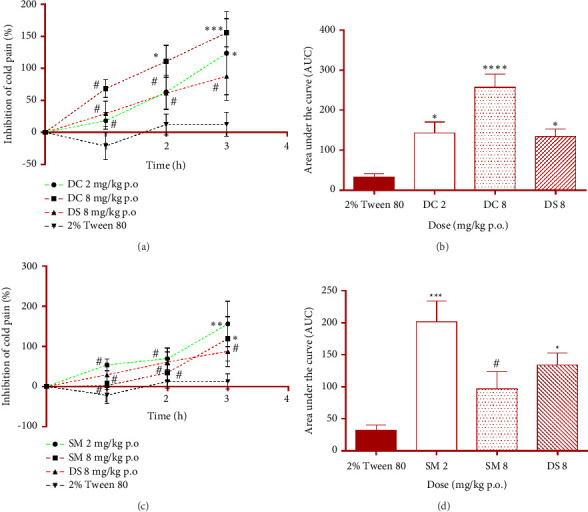
Effect of daucosterol (DC), *β *-sitosterol 3-myristate (SM), and diclofenac sodium (DS) on cold-induced pain in rats' paw on time-course curves (a) and (c) and the overall cold pain response calculated as the net area under the curves (b) and (d). ^∗^*p* < 0.05; ^∗∗^*p* < 0.01; ^∗∗∗^*p* < 0.001; ^∗∗∗∗^*p* < 0.0001; or ^#^*p* > 0.05, (*N* = 5), compared to 2% Tween 80. Two-way ANOVA followed by Tukey's post hoc test for the time-course curve or one-way ANOVA followed by the Dunnett multiple comparison test for the overall analgesic response.

The effects of DC and SM on cold-induced pain are shown in Figures 5(a), 5(b), 5(c), and 5(d). Both DC and SM significantly (*p* < 0.05) and dose-dependently protected the rats against cold-induced pain by increasing their latency to react to ice touch.

The analgesic effect of DC at 8 mg/kg p.o. against cold-induced pain commenced in the second hour after administration. The overall analgesic effect of DC was significant (*p* < 0.05) at both doses of 2 and 8 mg/kg p.o., with analgesic activities of 143.00 ± 27.55% and 257.30 ± 33.13%, respectively. These values were significantly higher than the 33.22 ± 7.99% analgesic effect produced by the negative control (2% Tween 80) group. SM also offered an inverse dose-dependent analgesic effect, with SM at 2 mg/kg p.o., being significant (*p* < 0.05) compared to the negative control. The analgesic activities of SM at 2 and 8 mg/kg p.o. were 201.80 ± 32.39% and 96.79 ± 27.00%, respectively.

### 3.4. Molecular Docking Results

The results of molecular docking studies for DC, SM, and DS with TRPV1 are shown in Table 1. The DC–TRPV1 complex exhibited the most favorable binding affinity with a ΔG of −10.60 kcal/mol, compared to the ΔG values obtained for SM and DS. Analysis of amino acid residues involved in the binding interactions revealed that SER404 participates in the binding of all three compounds to TRPV1. In contrast, TYR401 was involved only in the binding interactions of the two natural compounds isolated from *C. erythrocarpos* (DC and SM), which both demonstrated more favorable ΔG values than the synthetic reference drug DS (Table 1).

## 4. Discussion

The carrageenan-induced paw edema model is the most commonly used in vivo acute inflammation model for assessing the efficacy of anti-inflammatory agents. This inflammation model is an extremely sensitive and reproducible assay used to evaluate NSAIDs and novel anti-inflammatory agents [22]. This assay is also employed to predict the mechanism of action of orally active anti-inflammatory substances due to its biphasic nature. The initial phase (0–1 h) is mediated by the excessive biosynthesis of inflammatory mediators such as histamine, serotonin, and bradykinin. The second phase is governed by the synthesis of prostaglandins (PGs) and various proinflammatory cytokines, including TNF-α, IL-6, IL-10, and IL-1β [23].

The results of our study showed that DC, administered orally at 2 mg/kg, produced significant inhibition (*p* < 0.05) of inflammation in rat paw from the first to the fourth hour (*p* < 0.0001) on the time-course curve. This indicates that DC inhibited both the initial and final phases of carrageenan-induced edema. Therefore, the anti-inflammatory activity of DC involves the inhibition of histamine, serotonin, and/or bradykinin in the first phase, in addition to PGs and proinflammatory cytokines in the second phase.

SM at 2 mg/kg p.o. also appeared to inhibit the carrageenan-induced edema in rat paw starting from the third hour, although the effect was statistically insignificant (*p* > 0.05) on the time-course curve. This indicated that, unlike DC, SM only inhibited the second phase of the carrageenan-induced inflammatory reaction. This suggested that SM inhibited PGs and proinflammatory cytokines to exert anti-inflammatory action but did not inhibit histamine, serotonin, and bradykinins. Furthermore, the classical NSAID, DS, used as the standard drug also significantly (*p* < 0.01) inhibited only the second phase of the carrageenan-induced edema but not the first phase (Figures 3(a) and 3(c)), as expected. Since DS is a standard COX-2 and COX-1 inhibitor.

The classical signs of inflammation are edema, erythema (redness), warmth, pain, and loss of function (stiffness and immobility). Carrageenan-induced edema in rat paws has been reported to reproduce these signs following subcutaneous injection [24]. Certain proinflammatory cytokines, such as IL-1β, IL-6, and TNF-α, which are also involved in inflammation-induced central sensitization, contribute to the development of contralateral hyperalgesia [25]. Moreover, decreased production of proinflammatory cytokines has been reported to reduce pain severity [26]. These previous discoveries suggest that anti-inflammatory substances that significantly inhibit the second phase of carrageenan-induced inflammation will also demonstrate significant analgesic action against inflammation-induced pain since the proinflammatory cytokines that initiate and sustain pain are mostly produced during the second phase.

We therefore investigated whether the isolated compounds could ameliorate the pain generated by the edema itself. The results showed that DC at 2 and 8 mg/kg p.o. produced significant (*p* < 0.001) inhibition of carrageenan-induced inflammatory pain with maximum effect at the fourth hour. SM at 2 mg/kg p.o. and DS also produced significant analgesic activity in this pain model. These results indicated that DC, SM, and DS might inhibit the production of proinflammatory cytokines such as IL-1β, IL-6, and TNF-α during the second phase of inflammation to lessen pain severity.

It has been reported that transduction of cold stimuli occurs through direct stimulation of cold-gated ion channels, blockade of voltage-gated Ca^2+^ ion channel activity, modulation of background K^+^ ion channel activity, and/or inhibition of Na^+^/K^+^-ATPase [27].

Generally, there are two classes of thermoreceptors that regulate cooling effects in the mammalian body: low-threshold thermoreceptors and high-threshold cold nociceptors that modulate mild cooling and extreme cold, respectively [28]. C- and Aδ-fiber low-threshold thermoreceptors are spontaneously active at neutral skin temperatures, but their firing frequency increases in response to small temperature drops and rapidly adapts once steady-state temperature is reached. High-threshold cold thermoreceptors are generally dormant at normal and low body temperatures; however, they fire impulses in response to extreme cold in the noxious temperature range [28].

Stimulation of TRPV1 initiates an influx of calcium and sodium ions, which leads to depolarization of the cell membrane, neuronal firing, and release of endogenous substances such as substance P, calcitonin gene-related peptide, and glutamate, which modulate pain transmission [29] and inflammation. Therefore, the inhibition of TRPV1 channels produces analgesic and anti-inflammatory effects. It has been reported that the majority of TRPV1 antagonists developed so far block direct stimulation of nociceptors by endogenous lipids, capsaicin, acidic medium, and heat [29].

The results obtained from the molecular docking experiments showed that the Gibbs free energy (ΔG) of the crystal complexes formed between TRPV1 and DC, SM, or DS were −10.80, −9.80, and −8.60 kcal/mol, respectively. This indicates that the isolated compounds might also inhibit TRPV1 to produce analgesic and anti-inflammatory activities. However, further in vitro and in vivo studies are needed to confirm this finding.

It has also been reported that the higher the -ΔG value obtained for a docking result, the better the degree of inhibition [30, 31]. Therefore, DC and SM might be better inhibitors of TRPV1 than DS. This was confirmed in the animal studies, where DC produced better anti-inflammatory and analgesic activities than SM and DS. Moreover, although SM showed better inhibition of TRPV1 in the molecular simulation studies (Δ*G* = −9.8 kcal/mol), its analgesic and anti-inflammatory effects were lower than those of DS in the animal experiments. This suggests that the primary mechanism of analgesic and anti-inflammatory action of DS operates through a different pathway other than inhibition of TRPV1.

DC is a naturally occurring steroidal saponin consisting of glucose as the glycosyl moiety bonded to C-3 of *β*-sitosterol, the aglycone. In contrast, SM, which is also a naturally occurring steroidal ester, has myristic acid bonded to the C-3 position of *β*-sitosterol instead of glucose as found in DC. The superior anti-inflammatory and analgesic activities demonstrated by DC compared to SM indicate that substitution of a glucose/monosaccharide at C-3 of *β*-sitosterol leads to increased activity compared to substitution of a carboxylic acid at the same position. This enhanced activity may be attributed to the presence of four hydroxyl groups in glucose, which improves dissolution and bioavailability of DC compared to SM, which lacks hydroxyl groups.

Previous studies have shown that DC possesses remarkable antioxidant and anti-inflammatory activities through inhibition of ROS, TNF-α, IL-1β, IL-6, IFN-γ, and macrophage infiltration. Additionally, it exhibits hepatoprotective activity against alcohol-induced liver damage via the p38/NF-κB/NLRP3 pathway and demonstrates cardioprotective activity [32–35]. However, this is the first study to report the analgesic activity of DC against inflammation and cold-induced pain and to describe its isolation from *C. erythrocarpos*.

Although the antiarthritic activity of SM and its isolation from *C. erythrocarpos* have been previously reported [15], this is the first study to investigate the acute anti-inflammatory and analgesic activities of SM, compare these activities with those of DC, and determine their effects on TRPV1.

Finally, further studies using in vivo animal models are required to address the limitations of this work, including safety/toxicity profiles and TRPV1 activation mechanisms of DC and SM.

## 5. Conclusion

This study has described the isolation and characterization of the steroidal saponin DC from *C. erythrocarpos* for the first time. It also showed that DC possessed significantly high anti-inflammatory and analgesic activities, higher than those of DS, against mechanical and cold-induced pain. SM, on the other hand, produced moderate anti-inflammatory and analgesic activities at the same doses as DC. Both compounds also remarkably inhibited the TRPV1 ion channel with very low -ΔG. DC and SM should, therefore, be considered for development into potential analgesic and anti-inflammatory drugs.

## Figures and Tables

**Table 1:** Results of docking of the ligands with TRPV1 ion channel transport protein 3j5p.

Names of ligand	ΔG (kcal/mol)	Amino acids	Crystal complexes formed
Daucosterol	−10.60	Chain B: TYR401 SER404 HIS410 TYR495 VAL508 ASP509 SER510 TYR511 SER512 LEU515 PHE522 PHE543ALA546 MET547 THR550 ASN551 LEU553 ARG557 VAL567 ILE569 GLU570 LYS571 ILE573 LEU574 ARG575 LEU577 CYS578 MET581 GLU692 ILE696 LEU699 GLN700 ILE703. Chain A: PHE587 PHE591 ILE661 LEU662 LEU664 ALA665 TYR666 ILE668 LEU669 LEU673	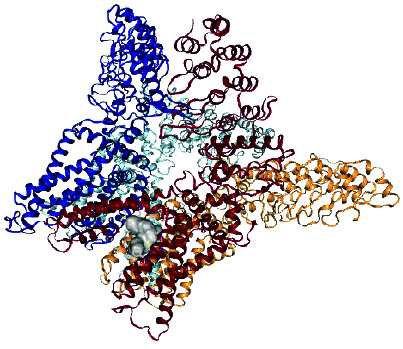

β-Sitosterol myristate	−9.80	Chain C: TYR401 SER404 PHE434 ASN437 VAL440 TYR441 LEU443 TYR444 ILE447 LEU480 SER481 SER483 GLY484 TYR487 PHE488 ARG491 GLN494 TYR495 PHE496 ARG499 PRO501 SER502 VAL508 ASP509 SER510 TYR511 SER512 GLU513 ILE514 LEU515 PHE516 PHE517 VAL518 PHE543 ALA546 MET547 THR550 ASN551 LEU553 TYR554 TYR555 THR556 ARG557 ALA566 ILE569 GLU570 LYS571 ILE573 LEU574 ILE696 GLN700 ILE703 THR704 ASP707 THR708. Chain D: PHE587 PHE591	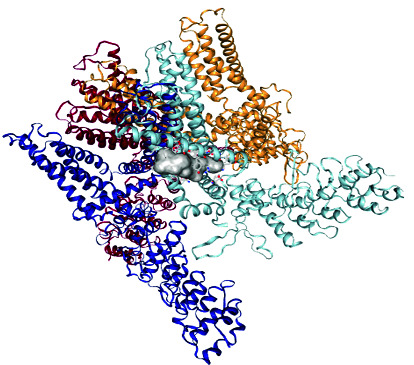

Diclofenac sodium	−8.60	Chain B: SER404 ARG409 HIS410 PHE434 ASN437 PHE438 VAL440 TYR441 LEU443TYR444 ILE447 LEU480 SER481 SER483 GLY484 GLY485 VAL486 TYR487 PHE488 ARG491 TYR495 ASP509 SER510 TYR511 SER512 GLU513 ILE514 PHE516 TYR554 TYR555 ARG557 VAL567 GLU570 LYS571 LEU574 ILE696 LEU699 GLN700 ILE703 THR704 ASP707	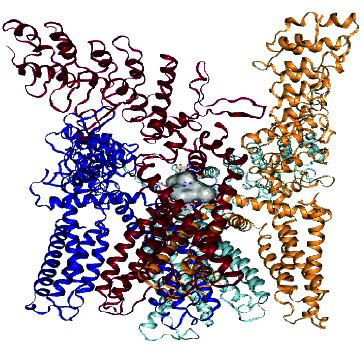

## Data Availability

Data are available on request from the authors.
